# Supplementary material for: Combined Membrane Dehumidification with Heat Exchangers Optimized Using CFD for High Efficiency HVAC Systems
Source: Membranes (Basel). 2022 Mar 22;12(4):348. doi: 10.3390/membranes12040348 (PMC9029657; doi:10.3390/membranes12040348)
Supplement: Supplementary file 1 [file membranes-12-00348-s001.zip › membranes-1610554-supplementary.pdf]

Supplementary Information

1| Meshing details and grid independence study

The following figure shows the meshing details of the 2D CAD model used in the CFD model for the study. The mesh used for the CFD study is shown in Figure S1. This mesh is modified to conduct a grid independence study whose results are shown in Figure S2.

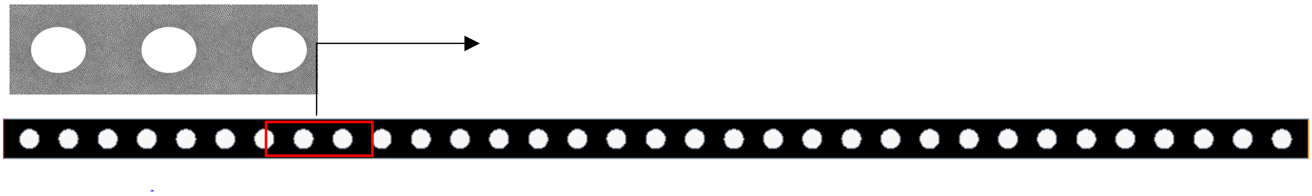

Figure S1. Meshing of base model used in the study.

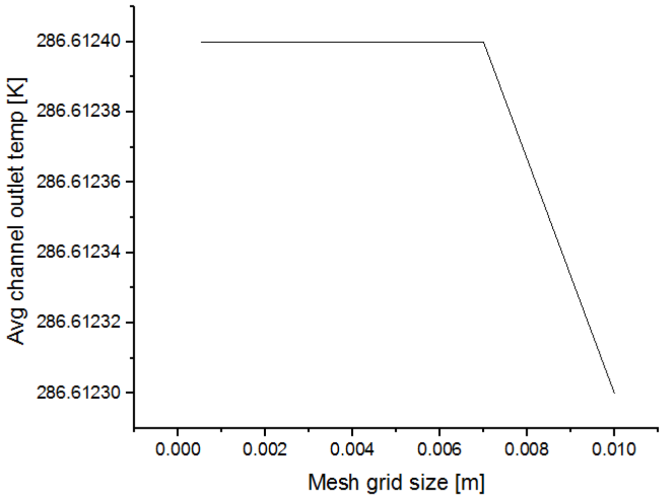

Figure S2. Meshing independence study performed on initial base model.

2| Convection coefficient and pressure drop CFD results

The following figure shows convection coefficient and pressure contour results obtained from the CFD study which is used to compare the analytical model.

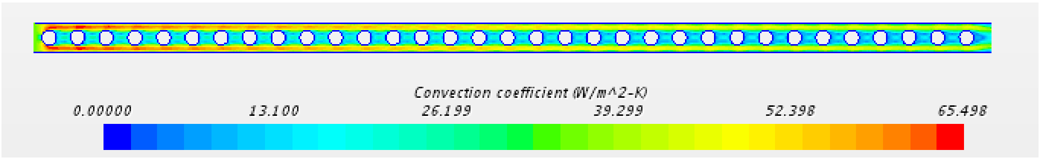

(a)

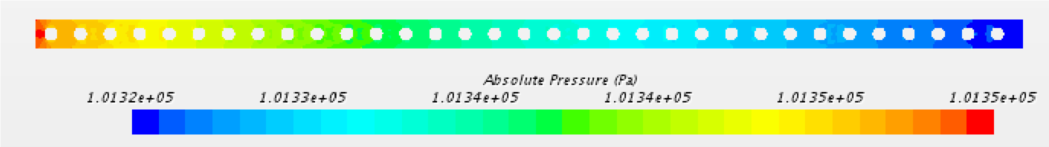

(b)

Figure S3. (**a**). CFD base model results for heat transfer coefficient results across the channel. (**b**) CFD base model results for pressure drop across the channel.

3| Analytical model for CFD comparison

The following figure shows the configuration of the inline coil bank for which the analytical model is used as a reference to compare the CFD results.

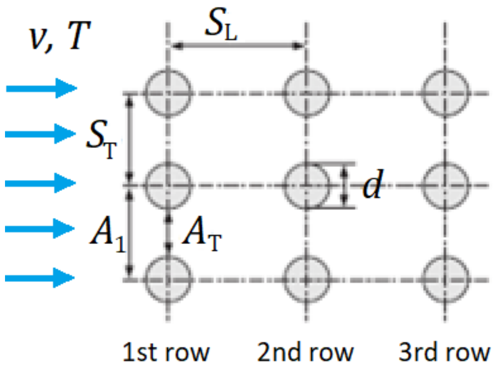

Figure S4. Geometry of analytical model used for comparison of the CFD model [51].

The temperature drop of a fluid flowing across an inline cylinder coil bank is calculated using the following equation:

$$T_E = T_s - (T_s - T_i)\exp\left(\frac{hA_s}{\dot{m}c_p}\right) \qquad \text{(SE1)}$$

Where T_E is the exit temperature after each column of coil bank, T_s is the surface temperature of coil banks, T_i is the inlet temperature of fluid prior to entering the coil bank, $\dot{m}$ is the mass flow rate of fluid through the coil bank, c_p is the specific heat of fluid , A_s is the surface area of all coils in the coil bank, h is the convection coefficient of the coil bank obtained from the Nusselt number correlation for an inline coil bank, which depends on Reynolds number, given by supplemental equation SE2;

$$Nu = 0.27Re_D^{0.63}\mathrm{Pr}^{0.36}\left(\frac{Pr}{Pr_s}\right)^{0.25} \qquad 1000 \le Re \le 10^5 \qquad \text{(SE2)}$$

Where Re_D is the Reynolds number at the hydraulic diameter of the channel, Pr is the Prandtl number at the average temperature of the fluid, given by the average of inlet and outlet temperatures of the coil bank. Pr_s is the Prandtl number at the surface temperature of the coil bank. The convection coefficient

obtained from the analytical correlation is 56 W/m^{2}K. The pressure drop of fluid flowing across coil bank is calculated using the following correlation

$$\Delta P = N_L f\chi\left(\frac{\rho V_{\max}^2}{2}\right) \quad \text{(SE3)}$$

where $V_{\max}$ is the maximum velocity of fluid in coil bank given by

$$V_{\max} = \frac{S_T}{2(S_T - d)}V \quad \text{(SE4)}$$

where S_T is the transverse pitch, which is the vertical coil spacing, and D is the diameter of the cooling coils, f is the friction factor obtained from Reynolds number versus friction factor graph for inline coil banks, and χ is the correction factor to account for the deviation of the coil arrangement in the channel from a square configuration. To calculate the temperature drop across the coil, the number of coils is considered a measure of length in the x direction. For example, a single coil refers to a distance of 6mm along the channel.

4| Effect of channel height and coil diameter on heat transfer performance

The effect of channel height and coil diameter on the temperature drop of the air is presented in Figure S5. Increasing the channel height decreases the temperature drop and thus the heat transfer effectiveness. This impaired performance is a result of thickening boundary layers as the channel height increases. Smaller channel heights also help to enhance the mass transfer of the system due to a higher mass transfer coefficient. Additionally, larger diameter coils with smaller channel heights result in high pressure drops. However, decreasing coil diameter also has a negative impact on heat transfer. Thus, there exists a tradeoff between the coil diameter and channel height, such that the d/h ratio should be chosen depending on a combination of requirements.

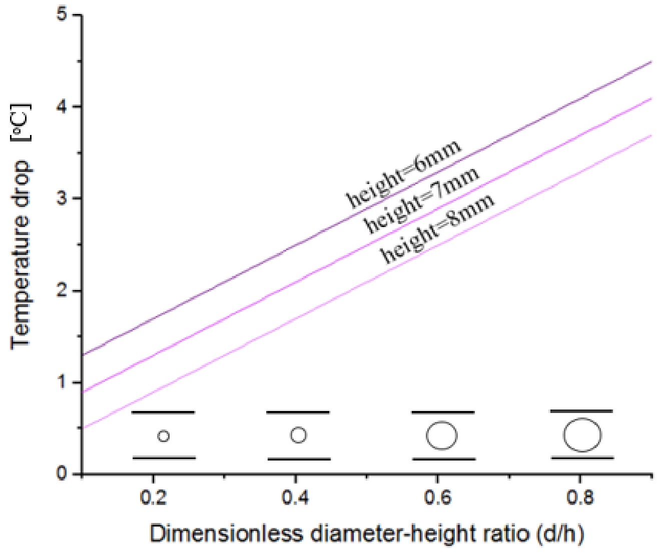

Figure S5. Temperature drop variation with diameter to height (d/h) ratio. T_{in} = 27°C, RH_{in} = 70%, v_{in} = 1m/s, N_{coils} = 33 coils, T_{coil} = 10°C, L x W = 0.2 m x 1 m, L_{cs} = h_{cs} = 0.006m, K = 5000 GPU. Coil diameter varies for a given channel height.

5| Impact of channel length and horizontal coil spacing on heat transfer

The impact of the horizontal coil spacing relative to the total channel length on the heat transfer is presented in Figure S6. At smaller horizontal coil spacings (L_{cs}), the flow does not have sufficient length to fully develop, resulting in an underdeveloped flow and smaller temperature drops (Figure S6, left). When the horizontal coil spacing is increased beyond a certain threshold, the fluid is fully developed and thus the temperature drop remains constant (Figure S6, right). The horizontal coil spacing also depends on other factors like coil diameter, Reynolds number, the channel height, and the membrane area to avoid condensation. For the current study, the ratio of horizontal coil spacing to channel length, for which flow becomes fully developed in the channel, is 0.025.

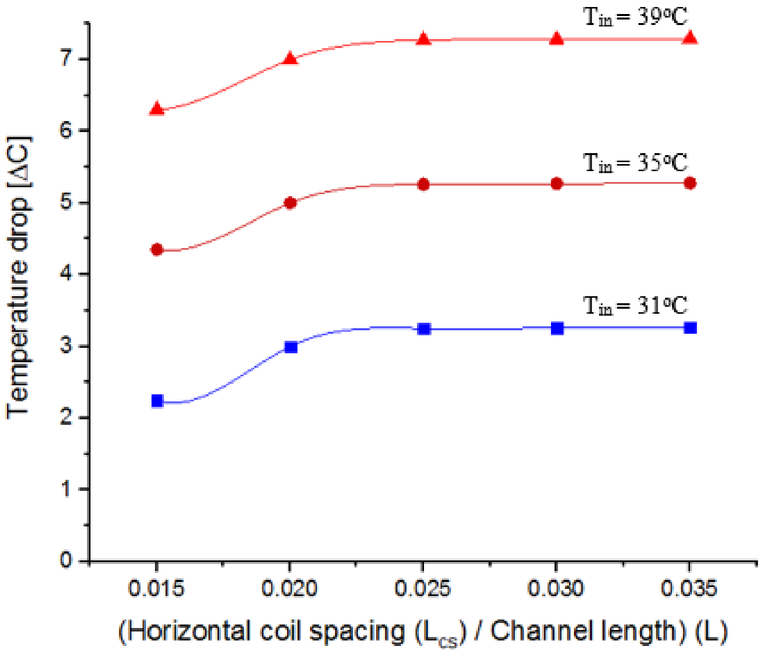

Figure S6. Channel length effect on heat transfer. Channel length needs to be sufficient to allow the flow to fully develop. RH_{in}=70%, v_{in} = 1 m/s, T_{coil} = 10°C, L x H=0.8m x 0.006 m, d= 0.003m, h_{cs}=0.006m, K=5000 GPU. Horizontal coil spacing is varied to obtain 33 cooling coils in the channel equidistant from each other.

6| Contour plot details - MATLAB

The contour plots from the results section are obtained with MATLAB after performing the necessary simulations of 50 data points for the required conditions with CFD. 50 data points was found to be sufficient and exhaustive enough to produce smooth contour plots that captured the varying trends.
